# Endotracheal Tube Kinking in the Prone Position during Pediatric Neurosurgery: A Case Report

**DOI:** 10.3390/children9101530

**Published:** 2022-10-06

**Authors:** Laura E. Gilbertson, Michael Morgan, Humphrey V. Lam

**Affiliations:** 1 Department of Anesthesiology and Pain Medicine, Emory University, Atlanta, GA 30322, USA; 2 Children’s Healthcare of Atlanta, Atlanta, GA 30322, USA; 3 Medical College of Georgia, Augusta University, Augusta, GA 30912, USA

**Keywords:** pediatric anesthesia, prone position, endotracheal tube kinking, posterior fossa surgery

## Abstract

Background: The prone position presents several concerns for the pediatric anesthesiologist, such as prevention of pressure related injuries, avoidance of undetected line infiltration, proper airway securement to inhibit unanticipated extubation, and limited access to the patient in critical events. However, the possibility of endotracheal tube kinking in pediatric patients is rarely discussed in the multitude of concerns about prone procedures. Here, we present a case report detailing the anesthetic management of a patient that experienced endotracheal tube kinking in the prone position during a posterior fossa mass resection. Our conclusion is that pediatric anesthesiologists must be cognizant of the possibility of endotracheal tube kinking in patients who are undergoing procedures in the prone position with significant neck flexion. We recommend using either an appropriately sized reinforced endotracheal tube or a nasotracheal intubation to decrease the potential of intraoperative tube kinking.

## 1. Introduction

Intraoperative airway obstruction can occur for a variety of reasons, requiring quick recognition, diagnosis of the source, and prompt treatment. Kinking of the endotracheal tube (ETT) is on the checklist of potential causes when experiencing an intraoperative airway obstruction, although it remains a relatively uncommon problem in anesthesia. However, procedures performed in the prone position present a significantly increased risk of such an event, while also presenting a more complicated process for correcting such obstruction.

Kinking of the most frequently used polyvinyl chloride (PVC) tube typically occurs in one of two areas [1]. One of these areas is at the connection between the anesthesia circuit and the ETT, and typically occurs due to the weight of the circuit, as distinct from the weight of the tube. Pediatric anesthesiologists are familiar with this issue, as small diameter ETTs are easily outweighed by the circuit, requiring stabilization. The other area that most frequently experiences kinking is intraorally proximal to the glottis, due to excessive neck flexion and/or softening of the ETT by increased intraoral temperature [2].

While pediatric anesthesiologists are often concerned about the prone position for surgical procedures, due to the known risk of accidental extubation, the potential for ETT kinking has been scarcely reported. In this case report, we present the anesthetic management of a pediatric patient who was undergoing posterior fossa resection and experienced kinking of the ETT while in the prone position. This manuscript adheres to the applicable EQUATOR guideline. The patient involved in this report had a written HIPPA authorization signed by the representative legal guardian for publication.

## 2. Case Presentation

A six-year-old patient weighing 21 kg presented for suboccipital craniectomy after exhibiting new onset nausea, vomiting, and hydrocephalus secondary to an obstructive posterior fossa mass. An uneventful inhalational induction was followed by administration of fentanyl, propofol, and rocuronium for intubation. Successful intubation occurred on the first attempt, utilizing a 5.5 mm cuffed oral Shiley PVC ETT secured at 16 cm at the teeth. A bite block was not placed following intubation. The patient was then secured in Mayfield pins and placed in the prone position. Anesthesia was maintained with sevoflurane combined with fentanyl for pain control and rocuronium for paralysis.

Approximately one hour after the start of surgery, the patient experienced a progressive increase in peak airway pressure from 17 to 39 cm H_2_O. A sudden, quick drop in end-tidal CO_2_ followed, which prompted us to take the patient off the ventilator and attempt manual ventilation. On initiation of this, a significant increase in airway compliance was detected. Auscultation revealed bilaterally decreased lung sounds. A 10 French soft suction catheter was passed down the lumen of the ETT to attempt removal of a potential obstruction, but it was unable to completely pass. Due to concern about a large obstructive blockage and progressive desaturation, the surgery and anesthesia team decided to have the incision quickly stapled and to reposition the patient supine.

**Figure 1:**
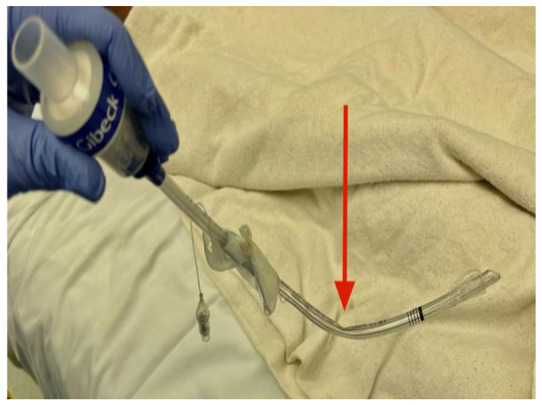
Endotracheal tube removed from patient with kink found at 11cm. Red arrow depicts site of kink.

Upon repositioning, the ETT was removed and found to have a large kink at 11 cm (Figure 1). Due to the finding of the kinked ETT, we discussed with the surgeons that minimal flexion would need to occur for the remainder of the procedure. The patient was uneventfully reintubated with a 5.5 mm ETT. The patient was then positioned prone with no significant changes in airway resistance after minimal flexion with the Mayfield system. The surgery was completed without further complication. The patient was extubated, and taken to the postoperative recovery unit. The patient remained stable on room air and was transferred to the intensive care unit for close neurological monitoring.

## 3. Discussion

This case report addresses the importance of swiftly identifying the potential for ETT kinking during prone surgical procedures in pediatric patients. Pediatric anesthesiologists have many complications to worry about when placing small patients prone, with careful attention paid to pressure injuries and appropriate positioning. From an airway standpoint, the primary concern is typically the high risk of ETT dislodgement secondary to traction on the ETT and decreased adhesiveness of tube tape from pooled secretions. In pediatric patients, there is relatively less safe room for the ETT to migrate before the tube dislodges. Because of the comparatively smaller diameter of pediatric ETTs, there is also the possibility of obstructive clots or material that can completely occlude flow. However, as this case demonstrates, there needs to be recognition of the potential for the ETT to become kinked during prone procedures.

ETT kinking in the prone position has previously been reported in a pediatric posterior fossa procedure, although it was not significant enough to require reintubation [2]. The increased incidence of ETT kinking during prone position procedures likely occurs due to a combination of factors. It has been demonstrated that more than minimal flexion at the atlantoaxial joint can cause enough mechanical bending to kink the ETT in the prone position. Additionally, as the intraoral temperature rises throughout the procedure, the typical PVC tubes soften and become more predisposed to kinking [3,4]. The reported events of ETT kinking have been noted to characteristically occur at least one hour after intubation. Our event was similar in the timing of the presentation and was likely due to a combination of increased oral temperature, PVC tubing, and excessive flexion from positioning.

There have been many different recommendations to help relieve and reduce the incidence of ETT kinking in the prone position. While a prophylactically placed bite block can prevent biting of the ETT, it normally does not reach far enough to prevent kinking at the oropharyngeal level. Ogden and Bradway reported the use of a Berman intubating airway, which is often used for fiber-optic intubations, to relieve an ETT kink in an adult prone craniotomy case [5]. However, the utility of this method in a pediatric airway may not be feasible. The utilization of a nasal ETT in pediatric prone procedures is preferred by some anesthesiologists, with the thought that it provides better stability from accidental extubation, avoids the potential for lingual injury, and decreases the incidence of kinking. As kinking of the endotracheal tube typically occurs at the junction where the oral and pharyngeal axes meet near the base of the tongue, the trajectory of the nasal tube allows it to transect this area at a less acute angle, thereby decreasing the potential for obstruction. However, providers should be aware that kinking may still occur if the patient is placed in significant flexion. Nasal intubation presents its own complications as well, particularly trauma to the surrounding nasal structures.

Sivapurapu reported successful replacement of a kinked PVC ETT with a reinforced ETT to relieve kinking [4]. Many commentators have discussed the advantage of a reinforced ETT for these procedures, as they are less prone to manipulation by heat. Nevertheless, they may still have the potential for kinking with increased degrees of flexion, such as occurs in posterior fossa procedures. In the pediatric patient, this technique may not always be practical, as the proper size of reinforced ETT may not be immediately available. In our case, we were not expecting to find a kinked ETT as our source of obstruction, and therefore we did not have a reinforced ETT ready for reintubation. As our patient required neck flexion for the procedure, a reinforced ETT would have been a good option to help decrease the potential of a complete obstruction of the ETT from kinking. Fortunately, our surgeon was amenable to using as minimal flexion as possible after reintubation and this helped alleviate further kinking episodes.

Caution must be taken with all of the potential oral options mentioned, as patients in the prone position are at increased risk for lingual edema. Oral airways, bite blocks, and reinforced ETTs, especially when used in small pediatric patients, can pose a significant risk for compression of the tongue after prolonged periods and should be monitored closely [6].

## 4. Conclusions

The prone position requires vigilant monitoring throughout the surgical procedure, due to the multitude of issues that can arise. As our case highlights, prompt recognition of ETT kinking as a cause of endotracheal obstruction is important. The obstruction may be significant enough to warrant stopping surgery, positioning supine, and reintubating. There have been a multitude of techniques suggested to either relieve or prevent ETT kinking. After experiencing this event, we advocate the use of an alternative airway securement technique, compared with the standard PVC oral tube. Depending on the anesthesiologist’s comfortability and the feasibility, either a properly sized pediatric reinforced ETT or a nasotracheal intubation would be preferable in a prone case requiring flexion for a prolonged operative period. While this does not absolutely mitigate the incidence of kinking, it decreases the possibility of total obstruction. Nevertheless, consideration of patient positioning and attentive monitoring throughout the procedure remain essential.

## Data Availability

Not applicable.

## Abbreviations

ETT—endotracheal tube, PVC—polyvinyl chloride.
